# Why do Public Debates Escalate? Trigger Points and the Moral Dynamics of “Hot Politics”

**DOI:** 10.1111/1468-4446.70115

**Published:** 2026-04-15

**Authors:** Linus Westheuser, Thomas Lux, Steffen Mau

**Affiliations:** ^1^ Max Planck Institute for Political and Social Science Göttingen Germany; ^2^ Humboldt‐Universität zu Berlin Berlin Germany

**Keywords:** affective polarization, moral economy, moral panic, moral politics, polarization entrepreneurs, political emotions

## Abstract

Escalating, emotionally charged, and moralized forms of controversy are a central feature of contemporary politics. Our study develops a framework for understanding how political debates between ordinary citizens become heated; why certain issues provoke particularly strong emotions; and how this affective potential is weaponized by “polarization entrepreneurs” in politics and the media. Based on an analysis of focus group discussions conducted in Germany, we identify recurring dynamics of affective escalation which we call *trigger points*. Trigger points are discursive breaches in which ordinary disagreement turns into affectively charged “hot politics”. Observing how debates escalate, the study argues that trigger points derive their affective potential from the violation of widely shared, implicit moral expectations. Specifically, we identify four widely recurring trigger points, each of which is rooted in a distinct set of moral expectations: “unequal treatments” (equality), “disrupted normality” (normality); “behavioral impositions” (autonomy); and “threatened boundaries” (control). Trigger points thus reveal a moral deep structure of public opinion. In this way, the concept helps bridge macro‐level diagnoses of polarized publics and micro‐level evidence showing that most citizens are only intermittently politicized. Trigger points illuminate how controversies between ordinary citizens can become charged with affects like anger, fear or disgust, without presupposing coherent ideologies or stable partisanship. More broadly, the framework offers a tool for analyzing wedge issues, perceived polarization, and the strategic emotionalization of politics by political entrepreneurs.

## 1 Introduction

This contribution studies moral dynamics behind the affective escalation of political debates. Previous research has shown that certain issues, situations, and frames provoke strong emotional reactions—such as anger, fear, or disgust—even among citizens not deeply engaged in politics (see Bakker et al. 2021; Webster and Albertson 2022 and below). These issues tend to be highly salient, identity‐relevant, and polarizing, leading to intense engagement and reduced openness to opposing viewpoints. Affectively charged, or “hot politics” contrasts with instances in which individuals engage with issues more dispassionately or analytically, relying on facts and reason rather than emotion. From an analysis of contentious group discussions between ordinary citizens in Germany, we develop a typology of discursive dynamics that “heat up” public debates. We call such moments of affective escalation *trigger points*, borrowing a medical term describing muscular tensions that are sensitive to touch and cause pain to shoot through wide areas of the body. Our analysis adds a qualitative political sociology angle to psychological research that has highlighted the political significance of moral foundations, emotions and codes (Ben‐Nun Bloom 2023; Graham et al. 2013; Haidt and Joseph 2004; Rozin et al. 1999; R. A. Shweder et al. 1997). In our contribution, we zoom in on how morality as a latent structure of public opinion becomes activated in contentious debates and how emotional forms of politicization unfold. We show that trigger points arise from violations of tacit moral background expectations; including standards of equality, autonomy, normality, and control. Trigger points lead to an emotionalization and escalation of public debates because they activate a socially shared deep structure of implicit moral expectations (Stinchcombe 1982).

Trigger points are a crucial dimension of contemporary mass politics, which studies have shown to increasingly rely on moralized and intuition‐rather than evidence‐based forms of reasoning (Aroyehun et al. 2025; Widmann and Simonsen 2024). Compared to technocratic expertise or the systematic belief systems that inform the stances of political insiders, trigger points stand for a more intuitive, moral, and affective form of political engagement (see Damhuis and Westheuser 2024). Understanding these dynamics elucidates why specific issues and issue frames find greater affective resonance than others in today's politics, especially among the majority of citizens not deeply engaged with politics (Krupnikov and Ryan 2022). Moreover, trigger points are an important tool used by strategic political entrepreneurs for purposes of political persuasion or for driving wedges into the electoral coalitions of opponents (van de Wardt et al., 2014).

The concept of trigger points may also help explain the phenomenon of “perceived polarization” or “pluralistic ignorance” (Ahler 2014; Fernbach and Van Boven 2022; Miller 2023) whereby citizens have the impression that the population of their countries are much more deeply ideologically divided than public opinion surveys suggest they are. For Germany, as for a large number of Western democracies, it has been shown that in the great majority of issues and countries, ordinary citizens' attitudes have *not* polarized in recent decades (Dochow‐Sondershaus and Teney 2023; Mau et al. 2026; Teney et al. 2024). Even in comparatively polarized countries like the U.S., most citizens hold belief systems characterized by low levels of coherence and “constraint” (Kinder and Kalmoe 2017), indicating that ideological polarization in the sense of systematically opposed belief systems is largely confined to politically informed insiders and engaged partisans (Krupnikov and Ryan 2022). Among ordinary citizens, incoherent views, as well as the avoidance and “selecting out” of politics are much more common than polarized political engagement (Eliasoph 1997; Groenendyk et al. 2024; Krupnikov and Ryan 2022; Peacock 2019; Settle and Carlson 2019). Yet on the other hand, there is a widespread *perception* among citizens that public debates have grown increasingly acrimonious, affectively charged, and polarized, and that an ever‐expanding set of issues is now fought in the uncompromising all‐or‐nothing mode typical for moralized and identity‐based conflicts (Achen and Bartels 2016; Bornschier et al. 2024; Fernbach and Van Boven 2022; Ryan 2017; Simonsen and Bonikowski 2022). In today's politics, mass depoliticization and an affect‐driven “hyperpoliticization” sit side by side (Jäger 2026; Pacewicz 2016).

In the following, we propose a way of connecting these seemingly contradictory findings. Drawing on the sociology of morality and studies of the moralization of politics, we explore why even among ordinary citizens not strongly committed to ideological partisanship or occupying a political middle ground, specific ways of framing political issues can lead to heated, controversial, relentless, and affectively charged political engagement. Through close observation of focus group discussions, we show that the recurring set of discursive dynamics which we call trigger points predictably turn up the heat in political discussions, amplify divisions, instigate disagreements and make it more likely that people feel pressured to take sides on polarized issues. We develop a typology of four typical trigger points that recur across a wide range of debates and settings. Arguments tend to become more heated wherever issues are cast in terms of *unequal treatments*, *disrupted normality*, *threatened boundaries*, or *behavioral impositions*. As shown below, all four of these types of trigger points stand for transgressions of implicit moral expectations of equality, normality, autonomy and control. Reconstructing the background assumptions implicit in trigger point dynamics reveals elements of a moral deep structure activated in moments of public controversy.

## 2 Theory: Morality and Affective Politics

The observation that the logic of political reasoning among ordinary citizens differs from that of elite politics is an old and fundamental insight of political sociology (Converse 1964; Gamson 1992). As Schattschneider (1975) pointed out, in real‐existing democracies only a very small portion of political issues are ever debated by the wider public; and among these, even fewer attract intense affective reactions. The ins and outs of zoning laws, fiscal policy, tax brackets, or municipal finances may provoke fierce arguments among experts, political insiders and directly affected citizens. But many of these debates do not reach wider publics, or tend to arouse only moderate affective responses when they do. This is different for what in political psychology is described as issues of “hot politics” (Bakker et al. 2021; Lodge and Taber 2013). Hot politics is defined by faster and more intense responses to political stimuli, be it in the sense of quick cognitive evaluations, or physiological responses measurable on respondents' skin and muscle tonus. Hot politics issues in recent times have included immigration and perceptions of a crisis of border control; allegations of rampant welfare cheating; concerns about the censorship of public expressions in the name of “wokeness”; as well as taxes and levies on fossil fuels or proposed lifestyle changes in the context of climate protection (see Mau et al. 2026 for a detailed discussion of the conflict structures these issues are embedded in).

The affective potential of issues like these is not necessarily proportional to their objective scope or the consequences of policies for people's daily lives. Many decisions with strong and immediate impacts on citizens' livelihoods, for example concerning finance regulation, pension schemes, or consumer protection laws, are not commonly debated in a “hot” register, while much smaller‐scale problems and infractions, such as welfare fraud, arouse extremely passionate responses. Often, these issues are of a symbolic rather than substantive nature. What previous studies have robustly established is that a crucial reason some issues trigger strong affective responses than others is their *moral* charge, that is its relatedness to moral assumptions or deeply held moral beliefs of ordinary citizens (Bakkær Simonsen and van Vree 2025; Garrett 2019; Ryan 2014). Morality here stands for a domain of reasoning centered around the evaluation of behaviors, relations, and groups as right or wrong, just or unjust, deserved or undeserved, and worthy or unworthy (see e.g. Hitlin and Vaisey 2010). People have a sense of justice and appropriateness which defines what they find morally plausible and acceptable. As Sayer (2005) theorizes, morality is affectively “hot” because it derives from an emotional process of relating to the flourishing and suffering of others, and because moral evaluations directly impact self‐understandings and feelings of worth. Understood in this way, morality is a central dimension of public opinion and the dynamic of political conflicts (Ben‐Nun Bloom 2023).

Two particularly important strand of research on the links between morality, politics and affect are Moral Foundations Theory and mapping of moral emotions and codes associated with the CAD (community, autonomy, divinity) triad (Graham et al. 2013; Haidt 2013; Rozin et al. 1999). Moral Foundations Theory is a program for explaining the intuitive and affectively charged character of everyday moral judgment, as well as systematic heterogeneity in the moral grammars that individuals and groups bring to political reasoning. Moral judgments are thought of as rapid, automatic intuitions, with deliberation operating largely ex post, for example as a form of justification or motivated reasoning (Haidt and Joseph 2004). Painting a picture of moral pluralism, the approach identifies sensitivities centered on different sets of moral foundations that structure what is experienced as morally salient and emotionally urgent. Examples include care/harm, fairness/cheating, and authority/subversion (for a later extension of the approach see Atari et al. 2023). In political psychology, the most robust empirical signature of moral foundations theory are recurrent links between moral intuitions and ideological profiles.^1^ US liberals, for instance tend to prioritize the individualizing foundations of care and fairness, whereas conservatives also additionally draw on the binding foundations of loyalty, authority and sanctity (Graham et al. 2009). This also matters for public debates as studied here. Conflicts over welfare, discrimination, and humanitarianism, for instance, are more readily moralized via harm and fairness, whereas conflicts over national identity, social order, and sexual norms are more readily moralized via loyalty, authority, and purity.

In addition, studies of elite rhetoric and mass response show that political communication is often moralized through foundation‐consistent language, and that such moral rhetoric can structure agenda conflict by amplifying certain evaluative dimensions while muting others (Clifford and Jerit 2013). Adding to this, Rozin et al. ’s (1999) CAD triad—which builds on R. A. Shweder et al. 1997 work on the “Big Three” values of autonomy, community, and divinity—offers a more explicit mapping of moral codes to specific emotions: breaches of the moral code of community/hierarchy, according to this theory, will be answered by emotions of contempt; autonomy violations will prompt responses of anger, while divinity/purity violations will be answered by disgust. Parallel lines of research connect these differences to stable affective dispositions: disgust sensitivity, in particular, shows a reliable association with political conservatism and conservative voting, consistent with the hypothesized linkage between purity/sanctity concerns and disgust‐based moralization (Inbar et al. 2009).

In political science, too, there is a broad field of research on the moralization of public debates. Where earlier research defined “morality policy” more narrowly as pertaining to a small set of issues concerning “first principles”, such as abortion, prostitution, or euthanasia, newer approaches have looked more broadly at the processes by which certain areas of politics become moralized (Widmann and Simonsen 2024). Thus studies have looked, for instance, at how politicians use moralized speech to mobilize followers (Gerbaudo et al. 2023), how the moralization of attitudes undermines political compromise (Ryan 2017), how social media amplifies moralized messages (Brady et al. 2017), or how political and media actors often seek out moralized issues and strategically place them in the public arena as “wedge issues” (Jennings et al. 2020; van de Wardt et al., 2014); the latter being rhetorical strategies which intentionally attempt to split voters and to polarize the public in order to yield electoral benefits.

Studies have also shown that the association of controversial policies with morally (de) valued social groups drastically affects the way policies are formulated and their likelihood of finding popular support (Elder and O'Brian 2022; Ingram and Schneider 2015; Westheuser and Zollinger 2025). Here, findings from political science directly fuse with sociological insights on the moralization of group belonging and demarcation. Following a tradition of research on the constitution of groups by moral demarcations (e.g., of the normal and the deviant, the worthy and the unworthy) going back to Durkheim (1997 [1893]), studies of moral boundaries reconstruct the social logic of evaluative group distinctions (Lamont 2000). Similarly, Cohen (1972) work on “moral panics” showed that many moralized issues acquire their affective charge because “folk devils”—problematic outsider groups, or “polluting categories” (Douglas 2003 [1966]), associated with them—come to act as embodiments of larger threats to societal norms and values (Falkof 2020). Dynamics of moral panic are often manufactured by moral entrepreneurs in politics and the media, or other social fields like the police, government administration, or the medical and education professions (Jennings et al. 2020). Through moralized forms of problematization and charged discourses, issues are constructed as “social problems” that must urgently be acted upon (Becker 1963; Blumer 1971; Rosino 2021).

At the same time, the resonance public interventions by powerful groups can expect to find is conditioned by the tacit normative background expectations present in popular moral reasoning. These expectations have been shown to follow historically specific networks of normative ideas closely entangled with social structures of power and recognition, or moral economies (Götz 2015; Thompson 1971). While normative theories and political ideologies often seek to explicate and systematize principles of moral judgment, in ordinary political reasoning moral evaluations remain implicit (Honneth 1982), taking the form of tacit intuitions, gut feelings, or sets of moral repertoires actors draw on in various situations. Moral expectations can be viewed as deposited in the “background ideas” (Schmidt 2008) that motivate ordinary citizens' reactions to public debates (see also Abend 2014). Moral background ideas like these arise from a two‐way interaction between the spin of mediatized public debates and the normative dispositions and contested normative “common sense” of ordinary people (Damhuis and Westheuser 2024; Gramsci 1971; Hall 2017).

The concept of trigger points that we develop in this contribution seeks to bring together these different strands of theorizing and research and applies them to political debates. Trigger points focalize the situational emergence of hot politics, that is emotionalization and fast reasoning, from violations of implicit normative background expectations as theorized in the sociology of morality and moral economy. Trigger points, as will become clear in the empirical analysis below, stand for processes of affective escalation precipitated by perceptions of moral transgression. Where they are activated, matter‐of‐fact debates become emotionalized, people turn more implacable in their opinions and disagreement trumps potential consensus. Where political and moral psychology look at moral foundations on the level of stable individual dispositions and intuitions, we center our analysis on the interactive moral dynamics of debates between citizens and their roots in a supra‐individual social structure of the moral background.^2^ Like moral panics, trigger points can be manufactured and often occur as a feature of deliberately provocative or attention‐grabbing mediatized discourses (see also our elicitation method below). Political actors also actively employ trigger points as wedges issues in order to mobilize voters, to attract new constituencies or to dominate the public discourse. At the same time, the dynamics of trigger points tap into a relatively stable, though historically malleable structure of public sentiments that also limits the agency of political players. Not everything can become a trigger point—only those issues whose politicization is made to tap into underlying moral assumptions and frameworks. We focus on the dynamic interplay between the structure of moral background expectations and their activation in debates between ordinary citizens.

## 3 Data and Methods

In the analysis below, we develop a heuristic typology of trigger points on the basis of data gathered in six focus group debates (*N* = 42) conducted in Germany between November 2021 and May 2022, each lasting between 2 and 3 hours (the full dataset can be accessed at [anonymized]). The participants (see appendix A for a full list) were recruited based on education, income, and political views in three different constellations: Two lower middle class groups each consisted of six participants with lower incomes (net equivalent income less than 75% of the median, i.e. roughly 1500€/month or less) and lower degrees of formal education (lower secondary degree or less). Two upper middle class groups each comprised six participants with higher incomes (net equivalent income of more than 150% of the median, i.e. roughly 2900€/months or more) and higher education (university degree). Two so‐called “crisis groups” each consisted of nine participants recruited based on their political attitudes as revealed in a screener survey: using survey items on issues of migration, LGBT minority rights, and climate protection (see appendix B), in each group three participants were selected for their conservative views, three for progressive views, and three for indifferent or centrist views. For each of these three formats, one group was held in Berlin and comprised participants from the German capital, a non‐industrial hub of knowledge‐intensive work, and the rural East German state of Brandenburg; another group took place in the ex‐industrial urban center of Essen, again including rural dwellers from the surroundings of the city. Soft quotas were applied regarding gender, migration background, and age (as well as income and education for the crisis groups) to assure a diversity of participants. All focus groups were conducted by two moderators of the public opinion research firm Ipsos, with the authors of this study observing the discussion dynamics from behind a two‐way mirror.

The interview guide for the focus groups was designed to provoke the occurrence of trigger points: The longest part of the discussions was prompted by distributing a stack of 28 real‐life newspaper headlines printed on laminated cards (see appendix C for a documentation of the headlines). The headlines focused on questions of inequality, migration politics, gender and diversity, as well as climate change. The participants were asked to pick out, without much thought, those headlines that in their view represented controversial and hotly debated topics. These headlines were then collected and debated in the remainder of the focus group session. The aim of our experimental set‐up was, on the one hand, to allow the interviewees to decide as intuitively and unfiltered as possible which topics were particularly controversial in their eyes. On the other hand, we wanted to simulate how people mostly come across trigger points in everyday life, via media headlines, slogans and buzzwords. This also builds on the assumption mentioned above, of trigger points arising from the interaction between mediatized interventions of public actors and citizens' common sense.

Our interpretive strategy in the analysis was to collect moments in which debates became heated and then to identify discursive and rhetorical patterns recurring in these passages as well as the moments preceding them. To identify relevant segments for the analysis of trigger points, three researchers each went through all of the material and coded instances of strong disagreements in the group, of emotionalization, and of particularly emphatic and vehement speech.^3^ While searching the transcripts for trigger points, the coders also listened to the audio recordings of the discussion, paying attention to moments of louder and faster talk and quicker turn‐taking, which we took as indicators of emotionalization. We also focused specifically on statements indicating indignation (“how can this be?!”) and ridicule (“this is just bananas!”). After some segments were excluded after discussions between coders, we selected 200 relevant segments with a total length of approximately 24,000 words. We then summarized individual segments focusing in particular on the question what “trigger” prompted the sequence. Seeking out similarities across segments, we constructed a first heuristic typology (Kluge 2000) that was then further probed by asking what implicit moral expectations were required for the “trigger” to appear as a breach with a strong emotional valence. If the first of these steps was a “morphological” classification based on observable discursive dynamics (what is being said), the second one was a more interpretive step that sought to elucidate the implicit “horizon of meaning” of these dynamics (what normative stance is presupposed by speakers in order for their statements to be intelligible) (Gadamer, 1994 [1957]. 42). The approach of observing taken‐for‐granted structures through reactions to their being breached is inspired by Durkheim's (1973) observation that a norm is crucially defined by the sanctions attached to their breach (and in this vein also bears some resemblance to Garfinkel's (1967) breaching experiments), as well as Thompson (1971), Honneth (1982) and Moore (1978) explorations of feelings of injustice as indicative of an “implicit social contract” (see Westheuser and Beck 2025 for a similar analytical strategy). We chose an inductive approach to stay open for dynamics beyond those theorized in existing studies. After having identified the four types of trigger points reported below, our analysis reached saturation, with multiple candidates for a fifth type (such as anger over government incompetence or skepticism about the feasibility of changes) ultimately rejected as variants of one of the existing types or as insufficiently supported by our material. That being said, we would not at all preclude that further studies, especially in other national and social settings, could identify additional types of trigger points.

## 4 Results: A Taxonomy of Trigger Points

Two important findings are already inherent in this analytical strategy which we developed in interaction with the data: First, that when ordinary citizens “talk politics” (Gamson 1992), preferences are often not articulated in the form of ideological propositions but negatively, by flagging breaches of expectations which otherwise remain implicit^4^; and second, that in segments containing such breaches, a moral register of argumentation tended to go hand in hand with an emotionalized and “hotter” climate of debate. Trigger points describe a dynamic of affective rooted in perceptions of moral injury.^5^ In the following analysis, we present a typology of four typical trigger points (Table 1), focusing on the dynamics by which specific sets of breaches activate implicit normative background assumptions: *unequal treatments* violate moral background expectations around equality, perceptions of *disrupted normality* violate expectations of normality, *behavioral impositions* violate expectations of autonomy, and *threatened boundaries* violate expectations of control. All four trigger points appear recurrently across interview settings and in the context of a range of issues, as indicated by some of the exemplary topics of debate noted in Table 1. To some extent the dynamics of the four trigger points are also independent of ideological leanings in the sense that they appear in the discourses of respondents across the political spectrum, although we discuss below how right‐ and left‐wing political orientations also impact how trigger points tend to be framed.

**TABLE 1:** Taxonomy of four trigger points.

	Breach	Implicit normative expectation	Example issues from group discussions
Unequal treatments	Unequal treatment, unjust disadvantages or privileges, disrupted hierarchies of entitlement	*Equality* Equal treatment, meritocracy, reciprocity	Discrimination, “special rights” for minorities, unearned privileges of the rich and powerful
Disrupted normality	Loss of order, deviance, pollution, threats to identity through norm change	*Normality* Shared rules, habits, common sense	“Migrant crime”, lifestyles of the wealthy, trans women in women's sports
Behavioral impositions	Interventions into and disruptions of behavioral routines and expectations, fear of social stigma	*Autonomy* Self‐determination and freedom from direct authority in private sphere	“Woke censorship”, language reform, state‐endorsed vegetarianism, speed limits
Threatened boundaries	Perceived acceleration of social change, uncontrollable dynamics, inflation of expectations	*Control* Stability, controllability and predictability	“Open borders”, gender quotas, climate change, welfare demands

### 4.1 First Trigger Point: Unequal Treatments

The first kind of trigger point concerns a sense of injustice relating to the *unequal treatment* of groups, the typical expression of this type being: “that's not fair!” This type prominently includes moments where discussants decry discrimination against groups such as women or people of color, or where different rules apply to the rich and powerful than to the rest of the population. An expectation of formal *equality* in the sense of equal treatment stands in the background of this trigger point, although interestingly, this expectation can also be formulated in an anti‐egalitarian manner to attack the “preferential treatment” or “special rights” of minorities. Examples of perceived breaches include differential treatments and double standards, for example when women are paid less for the same work, when Germans with a migrant background are repeatedly asked about their “true” origin, or queer people are attacked for their sexual orientation. As mentioned, it is also the prerogatives of the rich and powerful that trigger a sense of unequal treatment, as in one instance of our group discussions where two respondents, a lower level clerk and a pensioner on state benefits, complain about the former boss of Deutsche Bahn corporation, Hartmut Mehdorn (a relatively iconic figure of economic elite power in the German context):

Torsten Maybe someone here still remembers Mr. Mehdorn?

Rüdiger Oh yea!

Torsten He drove three companies into the ground and produced nothing but crap. And still he made so much money. […] If I, as an employee, cause damage somewhere, then I can be held liable for it. But not him

Rüdiger Some cash in, and others are thrown out onto the street.

The fact that different rules apply to the rich than to the rest is also scandalized through stories of suffering, for example of pensioners who during the energy crisis “sit in the dark and freeze”, as a respondent puts it, while the executives of energy companies are celebrating. While these forms broadly align with an egalitarian political agenda, complaints about unequal treatments can also be cited against what are perceived as excessive demands for equality and inclusion, for instance when anti‐discrimination measures are portrayed as unfairly favoring minority over majority groups. Arguments like these too draw their moral‐emotional force from the perception that a norm of equal treatment is being breached. In this application equal treatment is defined via the generalizability of demands: entitlements are only legitimate if they apply to everyone. Thus groups like trans people, sexual and ethnic minorities may only demand what everyone else is entitled to, such as physical integrity or the recognition as bearers of rights. As soon as it is about recognition as a specifically different person, however, claims are rejected with the comment that it is “only a fringe group” (Walter) of “only zero point something” (Stefan), whose privileged treatment would violate principles of equality. One group participant thus indignantly rejects the idea that trans people should have special swimming times in municipal swimming pools, making an argument of equal treatment: “I think it's not fair. Look, I also look different, for example, because I am fat. But I can't just say, ‘I want special swimming times now because I'm fat, because I feel ashamed when there are slim women in the pool with me.’”

The first type of trigger points records how in a variety of contexts, discussants get agitated over breaches of an implicitly expectation of equal treatment. In terms of implicit normative expectations, the first type of trigger points draws its meaning from a specifically modern semantics of the equal value of all people (Rosanvallon 2011; Simmel 1995 [1901]). Whereas in the past the inequality of people was a self‐evident basis of the social order and defended by conservative and liberal thinkers, today everyone who wants to be considered a morally competent participant in the discussion must profess the fundamental ideal of equal treatment (Losurdo 2014; Robin 2011). What varies is how the expectation of equal treatment is interpreted. Depending on whether the moral breach is attributed to dominant or dominated groups, and whether it is prompted by discrimination or excessive demands, outrage over unequal treatments can both bolster and weaken egalitarian measures.

### 4.2 Second Trigger Point: Disrupted Normality

A second typical trigger point is prompted by perceptions of a *disrupted normality*. This type centers less on universal ideas of justice than about a common sense regarding the kinds of behaviors that can be considered as conforming, appropriate, justifiable, or tolerable (Geertz 1983). The normative background expectation whose violation indicates that a trigger point has been hit is that of a shared sense of normality. Precisely because ideas of normality usually remain implicit and tacitly assumed, they are so deeply moralized as to be de facto sacred (Goffman 1963). Emotive marks of this trigger point are both the visceral rejection of behaviors and “polluting categories” (Douglas 2003 [1966]) outside the bounds of respectability, as well as the fascination with figures who embody transgressions of the social norm (Becker 1963). The former is seen when Barbara, an interpreter, enters a discussion about of self‐identification as a basis for the legal recognition of gender with great vehemence:

Then a man can say, “I feel like a woman now” […] and that gives him permission to enter my changing room at the swimming pool and—let me be blunt—waggle his penis in front of my twelve‐year‐old niece. This opens the floodgates to rape, to pedophilia and so on. I am against this! I have a *big* problem with that.

In this case, outrage and emotionalization are not primarily driven by concerns about equality or differential treatment. Instead, what is central is a breach of the normal and acceptable conduct and division of social spaces, as enacted by the imagined intrusion of a “false” transwoman into a space reserved for women. Barbara paints envisions the encounter between a perfect victim, her young niece, and the polluting category of a trans pedophile taking advantage of legal protections (see Svatoňová 2021). Starkly stylized social figures embodying transgressions of the normality, acceptability, and safety like these (the theory of moral panics speaks of this as “deviancy amplification”) appear in many “hot” debate segments and across a wide range of contentious policy areas. In addition to sexual minorities, these included vividly depicted welfare scroungers living a life of luxury, the arrogant and wasteful super‐rich, high school students skipping class while pretending to demonstrate against climate change, SUV drivers, young thugs, and migrant members of criminal clans. With a view to the latter, Mareike, a left‐voting accountant complains:

All the shisha bars, they're firmly in the hands of Lebanese clans. They have their own laws and laugh about us. They drive Lamborghinis through the pedestrian zone while collecting [welfare] benefits. I think we can't tolerate that! We are a society after all.

Here, as in the previous example, the *moral* boundary of normality and respectability is quite literally linked to the boundaries and expectations of specific places (Cresswell 1996). The transgression that triggers outrage is found in figures and behaviors that are “out of place”, such as someone with a penis in a women's changing room, a benefit recipient in a Lamborghini, or cars in a pedestrian zone.

Such grotesque images can be understood as a figurative representation of what Douglas 2003 [1966] describes as a fear of *social pollution*. The tacit background of this type of trigger point is the moralization of a shared sense of normality, of things and people being in their place. This sense of normality is defined and continually reinstated by acts of repair and defense against potential threats occurring at the boundaries of the normal. The normative “center” of social life is defined by its boundaries; This is why transgressions and contact zones such as changing rooms, the bushes of parks or pedestrian zones, as well as social figures on the periphery of what is socially acceptable receive intense attention. Groups that are *socially* peripheral are *symbolically* central for the definition of the normal (Stallybrass and White 1986, 23f.). This makes it understandable why the emotionalization of debates about transgressive figures and the normal order of spaces and behaviors far exceeds their practical relevance for people's daily lives. Trigger points related to disruptions of the normal indicate the potential precarity and vulnerability of common sense and its boundaries; how the question of what is and is not part of shared normality directly touches on self‐concepts; and how threats to normality are met with a variety of affectively charged rhetorical techniques, including containment and defensiveness, insecurity, nervousness, or even violent assertions of the norm. As the boundaries of the normal often are not only morally but also libidinously charged, affective negotiations around “triggering” social figures of transgression tend to combine fascination and repulsion, stimulation and disgust (see also Rozin et al. 1999; Rubin 1984).

### 4.3 Third Trigger Point: Behavioral Impositions

The third type of trigger point revolves around concerns for private autonomy linked to behavioral impositions. What respondents here perceive as an overstepping of a red line that warrants outrage is that authorities or other citizens are illegitimately trying to tell them how they are to live, think, speak, eat, or move. “Suddenly you have to…”, “Nowadays you're not even allowed to…” are typical phrases indicating this type of trigger point. What becomes tangible in the temporal aspect of these phrases—something that used to be acceptable is now being banned—is that very often perceived impositions are linked to changing routines and behavioral expectations, or challenges to established moral repertoires. Shifts in linguistic conventions and sensibilities are a key area where these types of changes are felt and commented on. Here, many respondents reject behavioral changes perceived as impositions even if these changes are broadly in line with their stated values, a pattern social psychology calls *reactance (Brehm* 1966 *; Rosenberg and Siegel* 2021 *).* Conservative‐voting Berlin police officer Ilko, for instance, remarks about sexual minorities:

You should let them live. For me personally it only gets difficult when it affects my own life. […] Everyone should get whatever operation they want, everyone should love whoever they want. But I don't want this very small minority to rub it in my face every day, and I don't want to have to ask everyone what their pronouns are.

“It gets difficult when it affects my own life” is a key phrase for this type of trigger point: While a permissive tolerance is condoned in the public sphere of politics and in questions of abstract rights, reforms and normative expectations targeting everyday behaviors are perceived as absurd, burdensome and paternalistic. The normative background assumption of this type of trigger point then is a negatively defined *autonomy* of the private individual and its personal conduct. Such negative freedoms, in the sense of a right to non‐interference by others in the private actions of individuals, can be understood as a foundation of the liberal tradition of individual rights (Berlin 1969). Cross‐cultural moral psychology has shown that this expectation of a private sphere which one may legitimately defend against the influence of others tends to be more pronounced in the individualistic societies of the rich West than in other, more collectivistically oriented societies (R. Shweder 1991). In everyday consciousness, the negative “freedom from” comes in a number of different forms, which have in common that they insist on not being obliged by certain newly arising behavioral norms and expectations. Again, this is a generic form appearing in the context of a number of different issue arenas. In debates about climate policy, for example, it is often the consumer's autonomy and freedom of choice that is vehemently defended against impositions:

I don’t like how we are being forced into e‐mobility. It’s just like with the vaccinations. And by the way, I'm not against vaccinations, I'm vaccinated. But we are being *forced*! That is the problem, that I'm being forced by the state to buy a vehicle that I don't even want.

This type of outrage is not about equality or normality, but rather revolves around the legitimate boundaries of paternalism, moral coercion and obligation (see also Amlinger and Nachtwey 2024), as well as the problematic relationship between changing social conventions and the expectation of individual private autonomy. A typical example is the perception that a failure to comply with ever‐changing norms of non‐discriminatory speech will instantly lead to accusation of right‐wing extremism. In the following segment, Walter illustrates this by reference to the now widely rejected name of a sweet which contains the German equivalent of the n‐word:

Walter You always have to be careful when you say things. So that you don't…

Stefan …get put into a box.

Walter … so that certain people don't take it the wrong way. Because then you're quickly pigeonholed as a right‐winger. A Nazi.

Birgit Yes, exactly.

Walter I still remember how I used to go into a German grocery store to buy a chocolate marshmallow, [and I would say:] “one (N‐) please.” And there were no problems at all. Today I would have to be *ashamed* of that! And if you question that critically or make a joke about it, then you immediately end up in a corner where you don't want to be.

The trigger point of behavioral impositions represents a flip side of the trigger dynamic labeled as disrupted normality. Where the latter was about sanctioning the transgressions of others, here the triggered find *themselves* unwillingly on the side of the transgressors and of those being sanctioned by a changing normative consensus. In this sense, the trigger point is not so much about literal prohibitions against certain behaviors, such as legal punishments, but on changes in the rules of the interaction order and its logics of shame, disapproval, and stigma. This is illustrated in the continuation of the segment, where an interesting misunderstanding arises when one respondent, Georg, interrogates the idea that “you are not allowed to say anything anymore”:

Walter [shrugs] You're not allowed to say anything anymore, am I right?

Birgit No, you're not.

Sven That's the way it is nowadays. No matter what the topic.

Georg [turning both palms up] But I hear you say it, so it seems that you are allowed to say it! I mean, you're saying it! So you *are* allowed to say it.

Birgit [loudly] No, no, but they changed that now! We're not allowed to say it anymore.

Georg But you *are* allowed to!

Walter [points at Georg] You can't just go and buy a (N‐) at the bakery! You'll get in trouble right away!

Besides the misunderstanding between a “legalistic” understanding of impositions and one centered on implicit everyday morality, this segment contains interesting hints about who people imagine as the actors of behavioral impositions. On the one hand, these are real or imagined fringe groups of moral entrepreneurs, such as militant vegans, who “want to impose their lifestyle on others” (Torsten). On the other hand, the sanction is often located at a higher level of authority, which defines the “official” rules of acceptable speech and is implicitly associated with professional superiors or decision‐making authorities: “*They* changed that”, as Birgit puts it. The tabooing of spontaneous, “normal” speech is interpreted as an intervention by patronizing social authorities. It can be assumed that the encroachment of socio‐political transformation processes into one's own everyday life is also met with angry reactance because it is others who are seen to set the pace and the direction of normative change. This raises important questions about the links between fears of a loss of autonomy and experiences of social agency.

### 4.4 Fourth Trigger Point: Threatened Boundaries

In the explanations of the previous types of trigger points, we have already come across a fourth discursive form that deserves special mention due to its prominence in group discussions: the fear of a *threat to important social and symbolic boundaries*, for example through a spiraling out of control of social processes or an escalation of demands. “What will be next?“ or “Where will this lead?” are typical reactions indicating that this type of trigger point has been hit. We already saw this logic in the passage quoted above, in which Barbara insinuates that allowing trans women into women's changing rooms would ‘open the floodgates to rape, to pedophilia and so on”. This passage and others following a similar pattern implicitly assume a trend in the course of which even seemingly small and insignificant changes form part of a much more comprehensive and potentially overwhelming process. Against this threat, respondents often articulate an expectation of *control*: changes are expected to occur in manageable steps and at a limited pace, avoiding the risk of changes cumulatively taking on a self‐accelerating dynamic. “I mean what's next? If now all marginalized groups—the dark‐skinned, pigment‐impaired, bald, obese—if they all want a special community for themselves?” (Walter). “I'm fine with it on the local level”, says one participant about granting non‐EU foreigners voting rights, “but I'm afraid that if they start there, where will they stop? You say yes one time and then it goes further and further. That's what I'm afraid of.”

The object of the fear of a dissolution of boundaries is a perceived inflation of demands. When one demand of the outsider groups is fulfilled, the next one immediately follows; you give away your little finger and lose your whole hand. In rhetoric, this is often referred to as a “slippery slope” argument, that is a form of argumentation that aims to show that although the changes one opposes might seem small at first, they will ultimately lead to massive and fatal consequences; absurd, excessive and unintended consequences. Such rhetoric has been a prominent streak of conservative political thought (Hirschman 1991; Robin 2011). Where in the 18th century authors like Joseph de Maistre and Edmund Burke conjured up a decline of civilization if civil rights were to be extended to the non‐propertied classes, later conservatives warned of the effects of the welfare state, women's suffrage or the dangers of equality for ethnic and racial minorities (Chamayou 2021; Crozier et al. 1975).

While the preceding triggers showed how the (perceived) breach of expectations of egalitarianism and normality leads to emotionalized reactions, the type discussed here represents provocations that arise from the frustration of implicit expectations of control, continuity, and stability. Sociopolitical processes such as immigration, ecological transformation or the diversification of recognized ways of life are ascribed an inherent momentum, in the course of which understandable measures are increasingly escalated to absurdity by escalating demands or spill‐overs. At trigger points of this kind, a certain change fatigue seems to emerge. The respondents feel that they have already had to accept too many changes and are therefore allergic to further steps and the prospect of an ever‐increasing dynamization of society.

## 5 Summary and Discussion

In this study, we theorized and inductively studied typical dynamics of escalation in public debates, trigger points, as well as their roots in a substructure of tacit moral expectations. Our typology highlights certain generic dynamics, which can manifest in debates over a variety of issues. A crucial feature evident across the four types identified here is that trigger points are defined *negatively* through breaches and violations, or, put differently, by the perception that a red line has been crossed. All of the trigger points presented here follow a dynamic of violating implicit basic expectations. In trigger points, the normative consensus of a group or society as a whole receives its contour from the margins, that is through the common rejection of various types of transgressions. “That's going too far” is the near‐universal universal formula in which trigger points find expression. This formula highlights collisions between a fact, statement or opinion and latent normative standards felt to be “self‐evidently” valid.

In the case of the trigger point “unequal treatment”, transgressions of this red line manifest as particularly blatant moments of unfairness and discrimination, or instances where groups are perceived as gaining undeserved advantages, for example, by evading obligations, violating norms of reciprocity, or bypassing the order of social entitlements. These provocations are all based on a modern egalitarian‐meritocratic moral economy centered on *equality* norms. In contrast, trigger points based on expectations of *normality* arise when others overstep acceptable boundaries or display ideas of normality that clash with citizens' deeply held self‐perceptions or common sense. These trigger points are driven by concerns about losing the order of a “normal” everyday world. In the case of the trigger point of threatened boundaries, it is the violation of a tacit expectation of *control* that triggers a hot response. In public debates, this occurs when people feel that things are spiraling out of control and threatening to unleash chaos. Lastly, perceived encroachments into a sphere of private self‐determination and negative freedom rights can also act as a trigger. This leads to a breach of the implicit expectation of *autonomy*, which is often triggered by changes in behavioral norms and the interactional structure of social disapproval and sanctions that support them.

Although resulting from an inductive analysis with a focus on interactive dynamics of political debates and the supra‐individual normative structure of the “implicit social contract”, rather than the individual dispositions studied in social psychology, our typology can be linked to established schemes from moral psychology: Trigger dynamics around unequal treatments can be connected to moral foundations of fairness and cheating (or even the moral foundation of equality posited in newer iterations of the theory, see Atari et al. 2023); behavioral impositions echo the liberty/oppression foundation; disrupted normality and threatened boundaries resonate with sanctity and authority foundations.^6^ To what extent different types of trigger points can be linked to discrete moral emotions, in the sense of the CAD framework, would merit a more systematic investigation than our material allows for. As a starting hypothesis guiding such studies, one could assume that unequal treatments, as breaches of norms of communal fairness and hierarchies of deservingness, should elicit contempt; behavioral impositions, as violations of autonomy, should provoke anger; while disruptions of the normal and threatened boundaries should invite reactions of disgust. The elective affinities between our inductive framework and extant work in moral psychology may signal that trigger points could be studied through surveys or experiments using variants of established instruments, such as the Moral Foundations Questionnaire.

What is certain is that the dynamic of trigger points is of the greatest political relevance. Trigger points can be instrumentalized by political actors as *wedges* to divide opponents' support coalitions (Hillygus and Shields 2008; van de Wardt et al., 2014). Directed at the public at large, trigger points can be used as a strategic tool to amplify or even altogether manufacture sociopolitical conflict. For *polarization entrepreneurs* using the specter of political disunity for purposes of political mobilization, trigger points are powerful tools for capturing citizens' attention, creating the impression of deep‐seated societal divisions, and thereby making it seem urgent and inevitable to choose one of the alternatives offered by these entrepreneurs themselves. The usage of wedge issues by political actors has been an important theme in political science. The study of trigger points, we believe, can clarify the moral substructure of many of politically observable wedge issues. Our non‐exhaustive typology in this contribution aimed to reconstruct important sociomoral underpinnings that explain why wedge issues work and trigger the desired reactions in citizens.

While comparative work on national and group differences in trigger points would certainly be welcome, we believe that our taxonomy can be applied across a wide range of social settings, providing a heuristic framework for identifying and categorizing the moral dynamics of hot politics beyond local specifics. Recent studies have shown that certain trigger points, such as anxiety over young male migrants or the presence of trans women in women's locker rooms, have been weaponized by political actors in different countries and have found resonance in very similar ways across a wide range of settings (see e.g. Frederiksen and Harboe Knudsen 2021). Understanding how trigger points diffuse across transnational networks of political entrepreneurs (Widmann and Simonsen 2024); how different media ecosystems further their dissemination and uptake (Van Bavel et al. 2024); and how the popular morality of different social groups conditions the effectiveness of trigger points (Damhuis and Westheuser 2024) would be promising avenues for further research. Two recent studies have already fruitfully employed the trigger point concept for large‐scale quantitative text analyses of social and print media debates (Antypas et al. 2025; Heide 2025). We believe that the study of trigger points could also be useful for shedding light on the dynamics of affective polarization (Iyengar et al. 2019). Speaking very generally, our assumption is that the more central a red line is to a group's self‐image, the more clearly it is crossed and the more one issue taps into different types of trigger points, the more likely it is that an issue will become the object of hot politics. The objects provoking trigger points in our material—Arab clan members, trans people, or the arrogant rich—have in common that they act as ciphers for comprehensive problems and discursive constellations. Trigger points touch on something deeper than the specific issue at hands. Our analysis explored important elements of this deeper normative dimension.

When public debates hit trigger points, normative background expectations and their contestation become apparent. How conflict flares up around these points thus illuminates the overall contours and politics of a moral “deep structure” (Stinchcombe 1982), which otherwise remains implicit and taken‐for‐granted. The norms and expectations forming this deep structure is rarely explicated in everyday life debates, as few citizens take the time to formulate a clearly contoured system of normative principles guiding their political reasoning. Neither systematic ideology, engaged partisanship or political expertise however are needed to react to perceived injustices and moral violations encountered in public issues. Consequently, such forms of moral affect and reasoning are particularly important for understanding the politics of that majority of citizens whose belief systems are less systematically molded by political ideologies (Converse 1974; Damhuis and Westheuser 2024; Elder and O'Brian 2022). The dynamics of trigger points are so effective because they are anchored in a substructure of moral convictions, self‐understandings, and patterns of justification inherent in an “implicit social contract” (Moore 1978). Where such basic moral expectations are violated, a strong affective charge is released. By observing the dynamics of defense and conflict that arise at trigger points, an entire moral cosmology of society can be revealed *ex negativo*. We believe that this moral substructure and the dynamics of its activation are central for understanding contemporary politics; particularly the simultaneity of mass depoliticization and the flaring up of controversial and polarizing issues. Much of today's hyperpolitical and affect‐laden political debates, we suggest, is not so much rooted in the clash of coherent political ideologies and polarized political camps as in strategically triggered affective reactions to the violation of ordinary moral expectations.

## Funding

Funding for this research was provided by the German Research Foundation (DFG).

## Conflicts of Interest

The authors declare no conflicts of interest.

## Supporting information


Supporting Information S1

## Data Availability

The full dataset is available at: Mau, Steffen; Lux, Thomas; Westheuser, Linus (2025): Triggerpunkte: Konsens und Konflikt in der Gegenwartsgesellschaft. Transkripte der Fokusgruppeninterviews [dataset]. Qualiservice, PANGAEA, https://doi.org/10.1594/PANGAEA.975394.

## Endnotes

^1^
Newer studies suggest that the causal arrow runs from ideology to morality (Hatemi et al. 2019).

^2^
For a discussion of the differences between dispositional and sociological‐relational accounts see also Westheuser 2025.

^3^
Besides trigger points that occurred in the focus group setting itself, we also coded those narrated by group participants as happening or having happened in other settings, for example family gatherings or the workplace.

^4^
This is true especially for parts of the population distant from politics, such as citizens with lower levels of formal education, members of the working class or other dominated groups (Westheuser and Beck 2025).

^5^
Mau et al. (2026, ch. 7) make a distinction between triggers (as the more proximate occasions for outrage) and trigger points (as the deeper underlying complexes of moral violation). We here elide this distinction for the sake of simplicity.

^6^
We are grateful to one of the anonymous reviewers for this very important observation.
