# Supplementary material for: Why do Public Debates Escalate? Trigger Points and the Moral Dynamics of “Hot Politics”
Source: Br J Sociol. 2026 Apr 15;77(4):701–12. doi: 10.1111/1468-4446.70115 (PMC13536507; doi:10.1111/1468-4446.70115)
Supplement: Supplementary file 1 [file BJOS-77-701-s001.docx]

# Appendix: ‘Trigger Points. The Moral Substructure of “Hot Politics”’

## Appendix A: Table of participants

| **Alias** | **Age** | **Occupation** | **Education** | **Place and Group Type** |
| --- | --- | --- | --- | --- |
|  |  |  |  |  |
| Linda | 47 | Cleaner | Secondary School | Berlin - Crisis Group |
| Sven | 51 | Real Estate Property Manager | University | Berlin - Crisis Group |
| Georg | 40 | IT Engineer | University | Berlin - Crisis Group |
| Walter | 59 | Public Servant | University | Berlin - Crisis Group |
| Birgit | 51 | Lower-level Clerk | Secondary School | Berlin - Crisis Group |
| Stefan | 46 | Industrial Middle Manager | University | Berlin - Crisis Group |
| Maja | 23 | Insurance Agent | University | Berlin - Crisis Group |
| Svea | 27 | International Development worker | University | Berlin - Crisis Group |
| Karlheinz | 73 | Retiree | University | Berlin - Crisis Group |
| Lisa | 39 | Drugstore sales clerk | Secondary School | Berlin - Lower Middle Class |
| Marco | 33 | Part-time Caterer | Secondary School | Berlin - Lower Middle Class |
| Torsten | 61 | Part-time Clerk | Secondary School | Berlin - Lower Middle Class |
| Rüdiger | 74 | Retiree | Secondary School | Berlin - Lower Middle Class |
| Anna | 30 | Part-time Clerk | Secondary School | Berlin - Lower Middle Class |
| Amar | 21 | Voluntary Social Service Year | Secondary School | Berlin - Lower Middle Class |
| Martina | 31 | Public Sector Economist | University | Berlin - Upper Middle Class |
| Charlotte | 24 | Architecture Student | High School (studying) | Berlin - Upper Middle Class |
| Ilko | 49 | Policeman / Criminal Investigator | University | Berlin - Upper Middle Class |
| Karin | 52 | HR Recruiter | University | Berlin - Upper Middle Class |
| Ludwig | 45 | Vocational College Lecturer | University | Berlin - Upper Middle Class |
| Goran | 58 | Lawyer / Business Consultant | University | Berlin - Upper Middle Class |
| Claudia | 61 | Civil Servant | University | Essen - Crisis Group |
| Barbara | 51 | Interpreter | University | Essen - Crisis Group |
| Hans Peter | 46 | Cook | Secondary School | Essen - Crisis Group |
| Mark | 48 | Logistics worker | Secondary School | Essen - Crisis Group |
| Dennis | 45 | Industrial Clerk | University | Essen - Crisis Group |
| Sandra | 56 | Nurse | High School Diploma | Essen - Crisis Group |
| Sabine | 57 | Clerk / Public Sector | Secondary School | Essen - Crisis Group |
| Christian | 35 | Soldier | High School Diploma | Essen - Crisis Group |
| Aliyah | 23 | Elderly Care Worker | Lower Secondary School | Essen - Crisis Group |
| Hilde | 67 | Retiree | Secondary School | Essen - Lower Middle Class |
| Fred | 38 | Landscaping worker | Lower Secondary School | Essen - Lower Middle Class |
| Marlene | 19 | Childcare Worker | Secondary School | Essen - Lower Middle Class |
| Natascha | 31 | Part-time Retail Worker | Lower Secondary School | Essen - Lower Middle Class |
| Josef | 45 | Mail Carrier | Secondary School | Essen - Lower Middle Class |
| Werner | 48 | Unemployed / Call Center Agent | Secondary School | Essen - Lower Middle Class |
| Carmen | 54 | Financial Accountant | Vocational college | Essen - Upper Middle Class |
| Mareike | 41 | Controller | Vocational college | Essen - Upper Middle Class |
| Ulrike | 35 | Primary School Teacher | University | Essen - Upper Middle Class |
| Jens | 61 | Social Worker | Vocational college | Essen - Upper Middle Class |
| Chiara | 28 | Event Manager | University | Essen - Upper Middle Class |
| Nico | 24 | Public Sector Consultant | University | Essen - Upper Middle Class |

## Appendix B: Screener survey for selection of participants

**Topic: Attitudes Towards Different Forms of Social Inequality (FINAL)**

**Recruitment Task**

- **2 face-to-face focus groups** of 2 hours each, with 6-7 participants each:
  - 1 group from the lower class (9 recruited for 6-7)
  - 1 group from the upper middle class or higher (7 recruited for 6-7)
- **1 face-to-face conflict group (CRISIS**) of 2.5 hours with 9 participants (12 recruited for 9), composed based on value orientations, organized as conflict groups
- **All participants must complete a quantitative questionnaire in advance** (approx. 30 minutes questionnaire duration)

**Recruitment Criteria**

- Gender mix (50/50)
- Age mix: 16-75 years, at least 1 participant in the 16-25 age group
- Educational mix (see specific quotas for groups)
- Employment situation and occupation mix
- Income mix (see specific quotas for groups)
- Urban/rural mix: 3 focus groups with participants from Berlin/Brandenburg, including rural surroundings
- For groups in Berlin: Mix of East/West socialization (aim for 50/50)
- No participant holds a political office
- **focus group** lower class:
  - Maximum secondary school graduation (*Realschulabschluss*/*Mittlere Reife*)
  - Lower income class < 75% of the median net equivalized income: Net equivalized income less than 1,470 euros
- **focus group** upper middle class or higher:
  - University degree (except students: at least one parent must have a university degree)
  - Upper middle class/high-income class > 150% of the median net equivalized income: Net equivalized income more than 2,940 euros
- **conflict group (CRISIS group):**
  - Educational and income mix
  - Composed based on value orientations: 3 left-liberal-cosmopolitan individuals, 3 conservative-traditional individuals, 3 moderate to indifferent individuals

**Introduction:** Good day. My name is ______________. We are currently conducting a study on the topic of "Social Inequality" on behalf of Humboldt University in Berlin. We would like to invite you to participate in our event, a group discussion that will take place on November 24/25, 2021. Your comments and views are very valuable to us and will have a significant impact on the results of our research. Your participation in the study could help better understand society in Germany and, thus, address social problems more effectively. This is an anonymous study. Your responses will be kept strictly confidential and will not be shared with third parties. This is not a sales event. Before asking you to participate, we would like to ask you a few questions to see if you fit our target group profile:

**0. Would you be interested in participating in this study?**

- Yes 1 → Proceed
- No 2 → Cancel

**1. GENDER** Please indicate your gender!

- Female 1 → Proceed
- Male 2 → Proceed

**!**50/50 per group

**2. AGE** How old are you? Please write: ________________

- Under 16 years 1 → Cancel
- 16-25 years 2 → Proceed to question 3a
- 26-35 years 3 → Proceed to question 5a
- 36-45 years 4 → Proceed to question 5a
- 46-55 years 5 → Proceed to question 5a
- 56-65 years 6 → Proceed to question 5a
- 66-75 years 7 → Proceed to question 5a
- 76 years and older 8 → Cancel

**!** Good mix, 1 participant per group between the ages of 16 and 25

**3a. EDUCATION** Are you currently attending a general education school (schools that do not end with a vocational qualification)?

- Yes 1 → Cancel
- No 2 → Proceed

**!** None of the participants is a student

**3b. SCHOOL LEAVING CERTIFICATE** What is your highest school qualification (without vocational qualification)?

- Left school without a diploma 1 → Proceed to question 6
- Lower secondary diploma *(Hauptschulabschluss)* or intermediate school diploma *(Mittlere Reife)* 2 → Proceed to question 6
- Upper secondary (*Abitur*) or specialized diploma *(Fachabitur)* 3 → Proceed to question 3c.

For all: Continue CRISIS Group

**!** Younger participants from the lower class have at most a secondary school leaving certificate/intermediate school leaving certificate

**!** No education quota for younger participants in the CRISIS group

**3c. HIGHEST EDUCATIONAL QUALIFICATION** What is your highest level of education?

- Completed university or technical university degree 1 → Proceed to question 4
- Upper secondary (*Abitur*) or vocational upper secondary *(Fachabitur)*, I am currently studying 2 → Proceed to question 4
- Upper secondary (*Abitur*) or vocational upper secondary *(Fachabitur)*, I am not studying (yet) 3 → Cancel

**[**Younger participants from the upper middle class are at least studying or have already completed a university degree.]

**4. PARENTS' HIGHEST EDUCATIONAL QUALIFICATION** What is the highest educational qualification of your parents or at least one of your parents?

- Lower secondary diploma *(Hauptschulabschluss)* or intermediate secondary school diploma *(Mittlere Reife)* 1 → Cancel
- Upper secondary (*Abitur*) 2 → Cancel
- Completed university or technical university degree 3 → Proceed to focus group of upper middle class

**! Younger participants from the upper middle class have at least 1 parent who has completed a university or university of applied sciences degree.**

**5a. SCHOOL LEAVING CERTIFICATE** What is your highest school qualification (without vocational qualification)?

- Left school without a diploma 1 → Proceed to question 6
- Lower secondary diploma *(Hauptschulabschluss)* or intermediate school diploma *(Mittlere Reife)* 2 → Proceed to question 6
- Upper secondary (*Abitur*) or vocational upper secondary *(Fachabitur)* 3 → Proceed to question 5b.

**!** All participants over the age of 26 from the lower class have at most a secondary school leaving certificate/intermediate school leaving certificate

**5b. HIGHEST EDUCATIONAL QUALIFICATION** What is your highest level of education?

- Completed university or technical university degree 1 → Proceed to question 6
- Upper secondary (*Abitur*)/vocational upper secondary *(Fachabitur)* is also my highest qualification 2 → Cancel

**!** Participants in the upper middle class over the age of 26 all have a university degree.

**!** CRISIS group: good mix of educational qualifications

**6. EMPLOYMENT STATUS** Are you currently...

- Full-time employed 1 → Proceed to question 7
- Part-time employed 2 →
- Self-employed 3 →
- On parental leave 4 →
- Retired 5 → Proceed to question 8a
- Student 6 →
- Employed on a minor basis 7 →
- Unemployed *(ALG I and II)* 8 →
- Housewife/househusband 9 →
- Other 10 → Cancel

**!** Good mix of employment situations

**!** At least 1 unemployed person in the lower class group

**7. OCCUPATION** What is your occupation? In which industry do you work? Please write:

Occupation: ___________________________________________________

Sector: ___________________________________________________

**8a. NET EQUIVALIZED INCOME / HOUSEHOLD SIZE** How many people live in your household (adults and children together)? (Shared apartments *(WG)* are considered individual households)

- Only myself 0 → Proceed
- 2 persons 1 → Proceed
- 3 persons 2 → Proceed
- 4 persons 3 → Proceed
- 5 persons 4 → Proceed
- 6 persons 5 → Proceed
- 7 persons 6 → Proceed

**!** Use the number of people in the household to calculate the equivalized income.

**8b. NET EQUIVALIZED INCOME / Monthly household net income** If you add everything together: What is your approximate monthly net income for your entire household, after taxes and social security deductions? Please include regular payments such as pensions, housing benefit, child benefit, State-funded student grant *(BAföG)*, maintenance payments, etc.!

Open query

Please write: Household net income:______________________________________________

**!** Please note: When selecting participants in the two middle class groups, the net equivalent income (except for very young members of the upper middle class) is decisive in addition to education; when recruiting, please note the respective net equivalent income according to household size on the basis of the attached Excel list for the stratum allocation and record the net household income stated in each case.

Median: EUR 1,960

Lower class group: less than 75% of the median net equivalent income: up to EUR 1,470

Upper middle class group: at least 150% of the median net equivalent income: EUR 2,940 or higher

CRISIS group: good income mix across the entire income spectrum

**9a. CITY/COUNTRY** Where do you currently live? Please provide your postal code.

Please write the postal code: ________________________________

**!** All participants: from Berlin/Brandenburg

! Approx. 30% from the surrounding rural area

**9b. CITY/COUNTRY** How would you describe the area where you live?

- A large city or a medium-sized city 1 → Proceed, urban
- A small town 2 → Proceed, rural
- A village 3 → Proceed, rural
- A cluster of houses 4 → Proceed, rural

**!** Please check with question 9a.

**10. SOCIALIZATION for Berlin** Where were you born? If before 1990, which part of Germany, GDR *(DDR)* or FDR *(BRD)*? If after 1990, in East or West Germany?

- GDR/East Germany 1 → Proceed, 50%
- FDR/West Germany 2 → Proceed, 50%
- Outside Germany 3 → Cancel

**!** Only for groups in Berlin: Mix of East/West socialization (please aim for 50/50)

**!** For people born after November 1989, you can aim for the relevant socialization information from their parents.

**!** No participants who were not born in Germany.

**11. VALUE ORIENTATIONS for CRISIS GROUP** **definition** On a scale of 1 to 5 (1 = strongly disagree, 5 = strongly agree), how much do you agree with the following statements?

|  |  | **Statement** | **1  strongly disagree** | **2**  **disagree** | **3**  **neither** | **4  agree** | **5  strongly agree** |
| --- | --- | --- | --- | --- | --- | --- | --- |
|  | **1** | **Foreigners living in Germany are an enrichment for German culture.** | conservative | conservative | indifferent | liberal | liberal |
|  | **2** | **It's good that there are wolves in German forests again.** |  |  |  |  |  |
|  | **3** | **Many people in Germany exaggerate their tolerance towards lesbians and gays.** | liberal | liberal | indifferent | conservative | conservative |
|  | **4** | **The authorities treat you like a number.** |  |  |  |  |  |
|  | **5** | **We risk our entire economic prosperity if we now put everything to the test because of climate change.** | liberal | liberal | indifferent | conservative | conservative |

**!** CRISIS: 3 liberal persons, 3 conservative persons, 3 indifferent persons per group

**!** Relevant items: 1, 3 and 5

**!** Liberal: “agree”/“strongly agree” for item 1 + “disagree”/“strongly disagree” for items 3 and 5 (at least one of these items should be strongly agree or strongly disagree)

**!** Conservative: “disagree”/“strongly disagree” for item 1 + “agree”/“strongly agree” for items 3 and 5 (at least one of these items should be strongly agree or strongly disagree)

**!** Indifferent: middle category for all three items (1,3,5)

**12. POLITICAL FUNCTION** Do you hold an office in a political party?

- Yes 1 → Cancel
- No 2 → Proceed

**!** No participant holds a party function or an office in a political party.

**13. DISCUSSION CAPACITY** How strongly do you agree with the following statement?

|  | **1  strongly disagree** | **2**  **disagree** | **3**  **neither** | **4  agree** | **5  strongly agree** |
| --- | --- | --- | --- | --- | --- |
| **I don’t mind discussing in a group of adults.** |  |  |  |  |  |

**!** No one is afraid to express their opinion, so it must be in the gray area.

**14. INCOME DISTRIBUTION ATTITUDE** How strongly do you agree with the following statement?

|  | **1  strongly disagree** | **2**  **disagree** | **3**  **neither** | **4  agree** | **5  strongly agree** |
| --- | --- | --- | --- | --- | --- |
| **Income should not only depend on an individual's performance. Rather, everyone should have what they and their family need for a decent life.** |  |  |  |  |  |

**!** No quota, please record

**15. RECORDING AND TRANSCRIPTION** For documentation and quality assurance purposes, the group discussion will be recorded and transcribed. The recording will be deleted in accordance with data protection regulations within 3 months. Do you agree with this procedure?

- Yes 1 → Proceed
- No 2 → Cancel

**!** All participants must agree to the recording and transcription of the focus groups.

**INVITATION** We would like to invite you to our group discussion on November 24/25, 2021, at N.N. o'clock in Berlin, at our studio at items. The focus group will last approximately 2 hours (or 2.5 hours for CRISIS). We do not want to sell you anything. You will be compensated ___EUR for your participation.

Are you interested in participating?

If yes, please provide your name, address, and phone number, and we will invite you.

**Recruiter’s Declaration:**

I, ............................................. (Name of the recruiter), hereby declare that the participant voluntarily provided all responses; I did not influence the participant in any way regarding their answers. I conducted the interview strictly according to the given guidelines and instructions. All information provided is truthful and accurate.

The interview was conducted in accordance with the above-mentioned instructions and the ADM guidelines for the implementation of market research studies.

The participant voluntarily agreed to take part in the group discussion.

Furthermore, the participant was informed that the group discussion would be audio and video recorded for research purposes. The participant was made aware that the client may, under the conditions of a confidentiality agreement (see below), be granted access to the recordings. The participant was assured that the recordings would be anonymized (i.e. no names, addresses, or similar personal information will be disclosed). The participant has given their consent to this.

I hereby confirm that I have communicated all of the above information to the participant and that the participant has agreed to all points.

**Signature:** ...................................................   **Date:** ....................................................

**Confidentiality Agreement**
(*To be presented to and signed by the client*)

In accordance with the guidelines of the ADM/BVM, the professional associations of market and social researchers in Germany, as well as the ESOMAR Code of Conduct of the Market Research Society, the anonymity of participants must be preserved when providing access to video recordings and interviews.

The client of the study, which was conducted in cooperation with IPSOS GmbH, hereby agrees to the following:

- The recording will be used solely for research purposes and only in line with the objectives of the study.
- The recording will not be passed on to third parties (including in the form of copies) nor presented to them.
- All individuals who are legitimately granted access to the recording will be informed of the contents of this confidentiality agreement and will be bound by the same terms.
- Any attempt to de-anonymize participants will be refrained from and actively prevented.
- The recording will be deleted no later than 3 months after receipt.

Ipsos GmbH cannot be held liable for any breach of this agreement, except in cases of willful intent or gross negligence.

## Appendix C: Headlines used as prompts for focus group discussions

As mentioned above, during the qualitative data collection, focus group participants were presented with a series of media headlines. These were laid out on the table as laminated cards in duplicate, and participants were asked to individually select two headlines within a relatively short amount of time (about three minutes) that, in their opinion, represented particularly controversial and hotly debated topics. The headlines were preselected based on our four arenas of inequality, with care taken to include a range of ideological perspectives and thematic aspects within each arena. Each arena featured seven headlines in the initial selection.

In the group session, participants were then asked one by one to read aloud which headlines they had identified as particularly controversial. These were then opened up for discussion (“And what do you think about this?”). This typically led to a lively and often contentious exchange of opinions, with specific arguments and rhetorical strategies. Together with the survey data, these discussions formed the basis for our reconstruction of the arenas. Our interest was often less in the specifics of the individual issues mentioned in the headlines (such as subsidies for cargo bikes), and more in the moral and argumentative repertoires they invoked, as well as the generalizable qualities that turn certain topics into trigger issues.

In the following section, we document the presented headlines and their translation into English.

**Top-bottom inequalities**

**Inheritance tax reform: An attack on wealthy heirs**

An inheritance tax reform could primarily burden medium-sized businesses

**Eco-transition only with redistribution**

High energy prices are a particular problem for the poor in Germany. The federal government can provide rapid relief by increasing the minimum wage, among other things

Hartz IV standard rates to rise in 2021

**Fourteen euros more to live on**

Hartz IV benefits are also set to rise due to the price increase. Social welfare associations consider this to be too little - and criticize the calculation method. By Cordula Eubel

CDU/CSU politicians call for unemployed

**Unemployed to sweep leaves in future**

Long-term unemployed people should be obliged to do community work in future, according to CDU/CSU politicians!

**Poverty risk for tenants on the rise**

At-risk rate was around 25 percent in 2019. Problems are likely to have worsened

**How inheritances cement inequality in Germany**

To those who have, shall be given: Germans are inheriting more than ever before, especially real estate assets. However, only part of the population benefits from this large flux of wealth."

Study on the minimum wage

**Millions earn less than 12 euros**

As at: 28.10.2021 14:20

According to a study, 8.6 million employees earn less than the twelve euros per hour that the SPD, Greens and FDP are planning as the future minimum wage. Women in particular would benefit from the increase.

**Inside-outside inequalities**

Migrants: Söder warns against uncontrolled immigration

Denmark: Migrants should work full-time for their social benefits

Refugees and the shortage of skilled workers / The long road to integration on the labour market

Refugees can help to overcome the shortage of skilled workers - this was said again and again in 2015. In the meantime, disillusionment has set in: many refugees receive social benefits or work in the low-wage sector. This is because the path to the labor market is long and arduous.

Do parallel societies exist in Germany?

Neighborhoods shaped by groups with a migration background, phenomena such as forced marriage or peace judges, are often cited as indicators of parallel societies.

**Migrant organizations call for local voting rights for foreigners**

The Federal Conference of Migrant Organizations advocates for local voting rights for foreigners living in Germany. This is one of the new proposals outlined in a draft for a Federal Participation Act presented yesterday in Berlin. The aim is to „strengthen our self-understanding as an immigration society,“ to take steps toward real participation, and to protect against racial discrimination, explained the conference spokesperson, Galina Ortmann.

Comments/Conflict between Poland and Belarus

**Hard heart, cynically heartless**

Stephan Fischer on the situation at the EU's external border between Poland and Belarus

“It is scandalous that the EU has had no answer to this since 2015”

The situation at the Belarusian-Polish border is intensifying. Thousands of migrants are hoping for a future in the EU. Migration researcher Ruud Koopmans warns of a situation similar to 2015—and proposes a radical shift in migration policy.

**Us-them inequalities**

Nationwide increase in attacks on queer people

Last year, the number of crimes against LGBTIQ* individuals in Germany rose sharply. The Greens criticize that the government is doing too little to combat homophobia and transphobia. BY INGA HOFMANN

GENDER-FRIENDLY LANGUAGE

Are state institutions allowed to change grammar? No!

By Olav Hackstein Updated on 08.11.2021 12:03

In the discussion about gender-sensitive language, there is often no distinction between the language system and its usage. Grammar does not discriminate. It has developed historically—and is simply misunderstood in the context of gender-neutral language. A guest contribution.

Diversity study

White men make TV

But women are catching up, at least in fiction. Diversity remains a major topic for the future of TV. By Joachim Huber

Trans*people in Berlin swimming pools

Soon they could swim for themselves

The Berlin trans*community is demanding designated swimming times in public pools. Next week, discussions will take place with the pool operators.

Panorama Project Kita-Suitcase

We want to question the concept of normality to some extent.

With an action plan, Saxony-Anhalt aims to create more tolerance for sexual minorities. A central component is the 'Kita suitcase' for educators. In daycare centers, the focus will no longer only be on traditional families.

A quota for trans people

As of 21.08.2021 11:36 AM

Argentina's authorities have introduced a trans quota of one percent. The LGBTIQ community celebrates. In the midst of the pandemic, the Catholic-influenced country is taking a pioneering role in gender equality

Why is co-motherhood being made so difficult?

More and more lesbian couples are fighting for both partners to be recognized as mothers upon the birth of a child. In fact, the so-called co-motherhood should already have been included in family law—if it weren't for the CDU

**Today-tomorrow inequalities**

The 1.5-degree target is unrealistic, dishonest and even dangerous

Most people believe that the 1.5-degree goal in climate protection is an inevitable result of science. However, it is a political goal, and one with many disadvantages: In the end, it might lead to exactly the disaster we are trying so desperately to avoid

Transport transition

Greens want a 1000 euro subsidy for cargo bikes

If it were up to the Greens, cargo bikes would receive more support—also for private individuals. They have now inquired how many bikes the Ministry of Transport has subsidized so far. The answer is disappointing

GERMANY

'FRIDAYS FOR FUTURE'

Teachers' Association criticizes skipping school for climate protests

Should students be allowed to skip school to demonstrate against climate change? In Berlin, even a teacher called for a school strike. Now, the teachers' association is speaking out. The political neutrality of the state is at risk, the association says.

The super-rich harm the climate far more than the rest of humanity

'A small elite is granting itself a free pass for the destruction of our climate': Oxfam highlights a strong socio-ecological imbalance in an analysis.

rbb - Radio Berlin-Brandenburg

Calculations by international researchers on climate change: Young people in Germany specifically threatened

When citizens sue against wind turbines
09.03.2020, 11:01 PM

How the dispute over wind turbines is slowing down the energy transition

"Unterleuten" in reality: In Brandenburg, the battle over wind energy has long become a rural-urban conflict.
By Werner van Bebber

Climate protection

Federal Environment Agency calls for tolls, speed limits and higher fuel prices

The Federal Environment Agency advocates for fundamental changes in the transport sector. Without massive efforts, climate protection will not succeed, says President Dirk Messner.

November 4, 2021, 11:29 AM / Source: ZEIT ONLINE, dpa, js / 807 comments
